# Assessing Upstream Determinants of Antibiotic Use in Small-Scale Food Animal Production through a Simulated Client Method

**DOI:** 10.3390/antibiotics10010002

**Published:** 2020-12-23

**Authors:** Zachary Butzin-Dozier, William F. Waters, Martin Baca, Rommel Lenin Vinueza, Carlos Saraiva-Garcia, Jay Graham

**Affiliations:** 1 School of Public Health, University of California, Berkeley, CA 94704, USA; zbutzin@berkeley.edu; 2 San Francisco de Quito University, Quito 170157, Ecuador; wwaters@usfq.edu.ec (W.F.W.); mbacab@alumni.usfq.edu.ec (M.B.); rvinueza@usfq.edu.ec (R.L.V.); hsaraiva@usfq.edu.ec (C.S.-G.)

**Keywords:** antimicrobial resistance, AMR, antibiotic resistance, ABR, simulated client, small-scale food animal, livestock, poultry, sales agent, One Health

## Abstract

Small-scale food animal production has been celebrated as a means of economic mobility and improved food security but the use of veterinary antibiotics among these producers may be contributing to the spread of antibiotic resistance in animals and humans. In order to improve antibiotic stewardship in this sector, it is critical to identify the drivers of producers’ antibiotic use. This study assessed the determinants of antibiotic use in small-scale food animal production through simulated client visits to veterinary supply stores and surveys with households that owned food animals (*n* = 117) in Ecuador. Eighty percent of households with food animals owned chickens and 78% of those with chickens owned fewer than 10 birds. Among the households with small-scale food animals, 21% reported giving antibiotics to their food animals within the last six months. Simulated client visits indicated that veterinary sales agents frequently recommended inappropriate antibiotic use, as 66% of sales agents recommended growth promoting antibiotics, and 48% of sales agents recommended an antibiotic that was an inappropriate class for disease treatment. In contrast, few sales agents (3%) were willing to sell colistin, an antibiotic banned for veterinary use in Ecuador as of January 2020, which supports the effectiveness of government regulation in antibiotic stewardship. The cumulative evidence provided by this study indicates that veterinary sales agents play an active role in promoting indiscriminate and inappropriate use of antibiotics in small-scale food animal production.

## 1. Introduction

Small-scale food animal production is a vital source of food security and income for families and individuals in low- and middle-income countries (LMICs) and provides opportunities for poverty reduction and improvements in gender equity [1]. However, it may also increase exposures to zoonotic infectious diseases among producers and their families, as the presence of animals living near the household increases human–animal interactions [2]. Furthermore, the use of antibiotics to promote growth, as well as treat and prevent disease, may augment the spread of antibiotic resistance (ABR) in animals and humans [1,3,4,5,6]. In humans, ABR is associated with 700,000 deaths each year, and without significant improvements in antibiotic stewardship, ABR could lead to 10 million deaths by the year 2050 [7]. Rural development organizations—including large international agencies such as the Food and Agriculture Organization (FAO) and non-governmental organizations (NGOs, e.g., Heifer International)—promote small-scale food animal production as a means of economic empowerment, but the degree to which they balance these benefits against detrimental impacts of zoonotic diseases and increased ABR remains an open question. Observational analyses suggest that small-scale food animal production improves child growth through improved access to nutrition but impairs child growth through exposure to zoonotic pathogens [8]. Infants and young children are particularly susceptible to exposures to zoonotic diseases due to their behaviors; observational research has shown that infants and young children often touch and consume soil that has been contaminated with animal feces [9,10]. Domestic animal feces have been found to be associated with a wide range of pathogens that can cause soil-transmitted helminth infections, trachoma, diarrhea, and growth faltering [11], and given the widespread nature of raising food animals in LMICs [3,8], this exposure pathway deserves greater attention.

The spread of clinically-important drug resistant bacteria between small-scale food animals and humans is well documented [12]. Antibiotics are widely used to improve animal health and prevent disease in both small- and medium-scale production as well as in large, industrialized units [13]. While antibiotics may, in some cases, have the potential to increase efficiency in food animal production, their use has also emerged as a global health problem due to their contribution to ABR [14]. Furthermore, the results of economic analyses have indicated that the small increase in efficiency produced by growth-promoting antibiotics is insufficient to offset the cost of the antibiotics themselves, resulting in a greater total cost of production [15]. The use of antibiotics in food animal production has been increasing since the 1950s, at the same time that farms have increased in size and animal density [16,17]. Consequently, the risk of ABR in farm animals is greater than before—as is the risk in human populations [16]. This problem is particularly serious in LMICs, where the use of antibiotics is often poorly and inconsistently regulated [18].

While the use of antibiotics in industrial food animal production is gaining attention, the impact of antibiotic use by small-scale producers is of growing concern. These producers often use antibiotics indiscriminately as a feed or water additive in order to promote growth, despite a lack of knowledge regarding the purpose, appropriate application, or dose of antibiotics [19,20]. All applications of antibiotics have the potential to contribute to the development and spread of ABR. Antibiotic stewardship interventions often target the indiscriminate uses of antibiotics, although therapeutic applications of antibiotics may also be problematic in the case of a viral infection or the use of inappropriate classes of antibiotics. 

In response to increasing ABR, certain classes of antibiotics have been identified as “last-line” drugs, as bacterial infections that are resistant to these antibiotics would have few alternative treatments [21]. Additional restrictions are often placed on veterinary use of these drugs in order to protect their utility in human medicine. In 2019, Ecuador announced a ban (effective January 2020) on the manufacture, sale, and use of colistin, a polymyxin class antibiotic, for use in animals [22]. Research in China has demonstrated that the removal of colistin from use in food animal production reduced the prevalence of colistin-resistant bacteria in humans, food animals, and food animal products [23]. Although interventions aimed at reducing antibiotic use in food animal production are associated with decreased antibiotic resistance in those animals, questions regarding the ability of governments in LMICs to enact and enforce these regulations have led to skepticism [24]. Evidence regarding the effectiveness of Ecuador’s ban on colistin for animal use can inform our understanding of government regulation as a sustainable tool for antibiotic stewardship. 

In a previous study, the authors conducted in-depth interviews with small-scale producers in highland Ecuador, where 84.5% of rural households and 28.5% of semi-urban households raised livestock or poultry [3]. Nearly half of the interviewees reported that antibiotic use was important for growth promotion and disease prevention, particularly for young livestock, and several participants claimed that antibiotic use prevented their animals from ever becoming ill [3]. No respondents mentioned specific human health risks for their families related to the use of antibiotics in the large-scale farms in the context of ABR, although several respondents expressed that consuming products from animals that have experienced antibiotic overuse could harm human health [3]. Although limited in scope, this pilot study suggested that antibiotic use is commonly practiced in small-scale food animal production and that this practice may be augmenting the spread of ABR in the community.

In addition to understanding the behaviors of small-scale producers, it is important to understand the attitudes, incentives, and behaviors of those who sell antibiotics. In Ecuador, as in many LMICs, antibiotics can be obtained over the counter without a prescription, which may lead to their misuse by small-scale livestock producers. Additionally, limited regulation and control of antibiotic use leads to a disparity between ideal and real-world antibiotic use practices [25]. Moreover, limited support, outreach, and oversight for veterinary sales agents may contribute to a divergence between recommendations and appropriate practices established by policy makers based on veterinary science.

Prior studies have examined the contribution of antibiotic use by small-scale livestock producers to ABR, but few have examined upstream influences on producers’ decisions to use antibiotics for their livestock [1]. In this context, “upstream” refers to the societal influences, particularly through veterinary supply stores, that may influence small-scale producers’ decisions to use antibiotic products [26]. This study aims to assess potential upstream determinants of antibiotic use in small-scale food animal production in five peri-urban neighborhoods east of Quito, Ecuador through household surveys and simulated client method (SCM) visits to veterinary supply stores.

## 2. Results

### 2.1. Survey Findings

Surveys were conducted with 117 households who owned small-scale food animals. Over half (54%) of the households owned fewer than 10 animals and approximately one-fifth (21%) had between 11 to 20 animals. Of the households with food animals, chickens were the most common (80%, not mutually exclusive) species owned, and 92% of the households with chickens owned fewer than 20 birds. Nearly one-fifth (21%) of the households that owned food animals reported using antibiotics with their animals within the last six months, with poultry (chickens, ducks, and quail) being the most common (88%, not mutually exclusive) type of food animal to receive antibiotics. Most households received information for how to apply antibiotics either orally (42%) or in a written format (38%; see Table 1).

### 2.2. Simulated Client Method Findings

Simulated client (SC) visits were made across the six neighborhoods to 38 veterinary supply stores in the growth promotion scenario and 40 stores using the disease treatment scenario. More than 80% of the stores carried antibiotic products. The most common professional role of the sales agent was employee, with other sales agent roles being store owner or veterinarian (see Table 2). 

#### 2.2.1. Growth Promotion Scenario Findings

When presented with the growth promotion scenario, 37% of sales agents immediately recommended antibiotics to increase growth (see Table 3). After the SC asked whether antibiotics would be effective for growth promotion, 61% of sales agents recommended antibiotics for this purpose. After restricting the sample to only include shops that carried antibiotics, these proportions of initial and final recommendations increased to 42% and 65%, respectively. 

In the growth promotion scenario, 66% of sales agents (25 of 38) recommended the use of antibiotics for growth promotion as their initial or final recommendation (see Table 4), although three of these sales agents did not recommend a specific antibiotic. Among the stores that recommended specific antibiotics, 9% (two sales agents) recommended a high caution antibiotic, 18% (four sales agents) recommended a medium caution antibiotic, and 73% (16 sales agents) recommended a low caution antibiotic [27].

Although there was no significant relationship between the presence of a veterinarian in the store and the sales agents’ initial recommendation (χ^2^ = 1.138, *p* = 0.286), in the growth promotion scenario, there was a significant association between the presence of a veterinarian and the sales agents’ final recommendation (χ^2^ = 4.316, *p* = 0.038) in this scenario (see Table 5). Whether the sales agent asked for additional information or appeared to be invested in the animals’ health was not significantly associated with initial or final recommendation of antibiotics in either scenario. 

#### 2.2.2. Disease Treatment Scenario Findings

Seventy-five percent of sales agents recommended antibiotics as their initial recommendation during the disease treatment scenario (see Table 3). After the SC asked about the efficacy of colistin (an antibiotic in the polymyxin class), 62% of sales agents recommended an antibiotic as their final recommendation. Few sales agents recommended the use of colistin, with only one out of the 40 stores visited (3%) in the disease treatment scenario offering to sell the drug. After restricting analysis to shops that carried antibiotics, the proportions of stores that recommended antibiotics were 85% and 76% for their initial and final recommendations, respectively. 

In the disease treatment scenario, 83% of sales agents (33 out of 40) recommended the use of antibiotics to treat the sick chickens as their initial or final recommendation (see Table 4). Of the agents that recommended antibiotics, 3% (one sales agent) recommended a highest caution antibiotic, 21% (seven sales agents) recommended a high caution antibiotic, 24% (eight sales agents) recommended a medium caution antibiotic, and 52% (17 sales agents) recommended a low caution antibiotic (see Table 4) [27]. 

## 3. Discussion

In addition to the development of a novel methodology to assess veterinary healthcare provider behavior, the results of this study suggest that antibiotics are commonly used in small-scale food animal production. Veterinary shop sales agents frequently recommend antibiotic use for growth promotion, disease prevention, and disease treatment for small-scale food animal production. Furthermore, these sales agents frequently recommend inappropriate antibiotic classes for veterinary use. This evidence supports the hypothesis that sales agents play an active role in promoting the use of antibiotics in small-scale food animal production. Given the association between veterinary antibiotic use in food animal production and antibiotic resistance, this information provides crucial insights to inform antibiotic stewardship [1,3,4,5,6].

Multiple influences can inform a veterinary sales agents’ antibiotic recommendation practices. Deficits in resources, particularly inadequate training, can prevent sales agents from providing effective treatment and appropriate antibiotic recommendations. This was supported by the finding that the presence of a veterinarian was associated with final antibiotic recommendation in the growth promotion scenario, indicating that the additional support and resources provided by an on-site veterinarian may influence sales agent antibiotic recommendation practices [28]. Inadequate regulation prevents policy solutions from guiding evidence-based antibiotic recommendation practices [29]. Peers and customers often influence a sales agents’ recommendation practices, which was demonstrated by the increase in the proportion of sales agents who recommended antibiotics in the final recommendation compared to the initial recommendation in the growth promotion scenario, although it is important to note that this study did not examine the determinants of producers’ requests for antibiotics [30]. Prior studies have found that small-scale producers often request veterinary antibiotics due to marketing campaigns directed at consumers, and veterinary sales agents may fear that they will lose customers if they do not offer to sell the requested antibiotics [25,30]. Finally, the profit motive often drives inappropriate antibiotic recommendation practices, as antibiotic sales provide income regardless of appropriate use [31].

The proportion of sales agents who recommended antibiotics for growth promotion indicates that they may be encouraging inappropriate antibiotic use. Any recommendation of antibiotics for growth promotion is inappropriate, as this encourages indiscriminate use. In the growth promotion scenario, 37% of sales agents immediately recommended antibiotics for growth promotion, and the proportion of recommendations increased to 61% when the SC asked specifically if antibiotics would be appropriate for that use. As indiscriminate antibiotic use for growth promotion has been identified as a target for intervention, this finding highlights a possible driver of this phenomenon [32]. While the use of veterinary antibiotics to treat disease is sometimes appropriate, one would ideally conduct diagnostics or antibiograms, which sometimes occurs in a high-income setting, or exhaust non-antibiotic options before resorting to antibiotics. Therefore, the fact that 75% of sales agents immediately recommended antibiotics in the disease treatment scenario remains a cause for concern. While this is the first study to observe authentic sales agent recommendation practices through a simulated client method, prior studies examining antibiotic use by small-scale food animal producers have found similar results regarding inappropriate and arbitrary antibiotic use [25,33,34]. For example, a study of small-scale poultry farms in Vietnam found that nearly half of antibiotic use occurred when no infection was present, and the majority of times that antibiotics were used to treat an infection, they were not effective [34].

In addition to the large proportion of sales agents who recommended the use of antibiotics, the classes of these products that were recommended are also problematic. Given that the sales agent did not establish previous antibiotic treatment and did not have access to any additional diagnostic information, the only appropriate antibiotic classes would be low caution, as these antibiotics are an appropriate first-line of treatment [27]. In the disease treatment scenario, nearly half of sales agents recommended an inappropriate antibiotic class and nearly one quarter recommended a high caution or highest caution antibiotic. Antibiotics from these two categories should be restricted from animal use in order to protect human health, and highest caution antibiotics are prohibited for veterinary use by the European Medicines Agency (EMA) [27]. The frequency of sales agent recommendations of these classes of antibiotics indicates a disparity between antibiotic stewardship guidelines and sales agent recommendation practices. This finding is consistent with a study that found that the most commonly used antibiotic class in Ecuadorian poultry production was fluoroquinolone, which is a high caution antibiotic class [30]. Furthermore, in the growth promotion scenario, 27% of sales agents recommended high caution or medium caution antibiotics. While no antibiotic is appropriate for growth promotion, the recommendation of inappropriate antibiotic classes for this purpose is particularly concerning.

In the growth promotion scenario, there was a significant relationship between veterinarian presence and recommendations for antibiotics after probing questions by the SC (χ^2^ = 4.316, *p* = 0.038), which suggests a relationship between the presence of a veterinarian and likelihood to concede to customer requests regarding antibiotics. This finding has potential implications on the relationship between the resources and training provided to sales agents and the likelihood that they would appease customer requests for inappropriate antibiotics. A 2019 study of veterinarians in Ecuador found that veterinarians frequently felt pressure to prescribe antibiotics, but it was not found that a significant relationship existed between veterinary antibiotic prescription practices and veterinary specialization or years of experience [30]. Hence, there may be a threshold of veterinary training that can enable sales agents to encourage antibiotic stewardship and resist customer requests for inappropriate antibiotics. Future studies should examine this relationship to pursue potential points of intervention to improve sales agent antibiotic recommendation practices.

The SCM results highlight the effectiveness of Ecuadorian legislation limiting the sale of colistin, which could serve as a model for other countries. Only one sales agent (3%) in the disease treatment scenario offered to sell colistin to the SC. It is important to note that these data were collected in late 2019, during the regulatory grace period following Ecuador’s announcement of a colistin ban, when stores were discouraged from selling colistin but were legally permitted to sell their remaining colistin inventory before it would be banned in January 2020. This delayed implementation led to concerns that there may be a short-term increase in colistin sale and use, given sales agents’ desire to liquidate their remaining inventory, but this evidence contradicts that concern. This finding provides compelling evidence that regulatory restrictions can effectively curb inappropriate antibiotic use in an LMIC.

The SCM described in this study is, to our knowledge, the first use of this methodology to determine veterinary recommendation practices. Observational and self-reported methods alone are insufficient to characterize sales agent behavior, as purely observational methods can be confounded by customer behavior, and self-reported methods can be vulnerable to various sources of bias [35]. The methods outlined in this paper may be useful in future studies because they can be replicated in a variety of settings to strengthen our understanding of veterinary health service provider behavior.

These findings have several implications for antibiotic stewardship policy. Given the frequency of veterinary sales agents’ inappropriate recommendations of antibiotics for growth promotion as well as the recommendation of inappropriate antibiotic classes, these sales agents may be an effective target for an upstream intervention to reduce drivers of inappropriate antibiotic use. Substitution of preventive and non-pharmaceutical interventions for antibiotics has been identified as a primary strategy in reducing the spread of veterinary ABR [36]. Potential interventions could apply a human-centered design approach to leverage sales agents’ self-perception of their role as veterinary healthcare providers to encourage this substitution. Rather than top-down regulation that would require monitoring and enforcement, this would facilitate a partnership between the private and public sector in which both groups recognize their role in preventing antibiotic resistance [31]. This emphasis on the sales agents’ role in protecting animal health as well as human health could provide motivation for sales agents to encourage non-antibiotic growth promotion and disease treatment interventions, including improved food quality, cleaner living conditions, and culturally-relevant herbal remedies [37]. 

### Limitations

There were several limitations to this study. From this information alone, one is unable to make causal statements regarding relationships between sales agent characteristics and recommendation practices or between antibiotic recommendation practices and actual antibiotic use. This study did not aim to compare antibiotic use or recommendation by scale of production. A mixed-methods study of antibiotic use in Ghana and Kenya found that medium- and large-scale poultry farmers’ antibiotic use practices were not correlated with animal service providers’ recommendations [29]. Furthermore, a mixed-methods study of antibiotic use across livestock systems in five African countries found that antibiotic use varied considerably based on the scale of production [38]. These cumulative findings support the hypothesis that antibiotic use may be related to the scale of food animal production. Future studies could explore nuanced differences between these groups, particularly in antibiotic use. For example, there may be a substantial difference in antibiotic use between those who rely on food animal production for profit and those who raise them for household consumption.

Sales agents may be less likely to recommend the use of antibiotics for certain species, and this study was not able to characterize sales agent behavior for different species of food animals. The results of the SCM scenarios, focused solely on chickens, may be different for different species of animals. Although, the survey respondents indicated that antibiotic use for small-scale poultry production was commonplace. Future studies could investigate this potential disparity in antibiotic recommendation practices by incorporating a range of food animals into SCM scenarios. Furthermore, future studies could assess the nuanced relationships between members of the supply chain to improve understanding of the influence of multiple stakeholders on antibiotic use in various types of food animal production.

## 4. Materials and Methods

The study was conducted between 2019 and early 2020 in six peri-urban neighborhoods east of Quito, Ecuador in the Andean highlands: Quinche, Yaruqui, Checa, Pifo, Puembo, and Tumbaco. These neighborhoods include 53 geographically defined areas where small-scale livestock production is common and have a total population of approximately 204,000 inhabitants. This study included SC visits to veterinary supply stores and household surveys. All participants were at least 18 years old and fluent in Spanish. This study and all procedures were approved by the Bioethics Committee at the Universidad San Francisco de Quito (#2017-178M), the Ecuadorian Health Ministry (#MSPCURI000243-3), and the Committee for Protection of Human Subjects at the University of California Berkeley (#2019-02-11803).

### 4.1. Simulated Client Method Procedures

Through random sampling of veterinary supply shops stratified by neighborhood to ensure proportionate representation, 40 veterinary supply stores were selected from a sampling frame of 81 stores previously identified and mapped in the six neighborhoods. Stores were eligible for selection if they sold veterinary supplies for food animal production. Each store was visited twice by trained simulated clients, with one visit being a disease treatment scenario and one visit being a growth promotion scenario (for the latter, two stores were not available). Investigators ensured an interval of two months between visits to each store and that each SC only visited each store once, in order to avoid undermining the simulated client interaction. Sales agents included male and female cashiers, store owners, and veterinarians. 

The SCM enables investigators to characterize authentic sales behavior through realistic but standardized client interactions. The methodology requires that the simulated client accurately represents a typical customer, is comfortable with the data collector role, understands the subject material, and has strong recall abilities [35]. This function was fulfilled by two Ecuadorian veterinary students who were trained and closely supervised by the field coordinator. 

Interactions lasted between 10 and 20 minutes and included a scripted combination of background information, requests for advice, and probing questions. In the growth promotion scenario, the SC explained that he or she has 25 chickens, which appear to be growing slowly compared to a neighbor’s chickens, and then asked for recommendations (see Supplementary Materials Instrument S1). Following the sales agent’s initial recommendation, the SC then provided standardized information regarding the health and living conditions of the chickens. In the event that the sales agent recommended an intervention to improve growth that did not require medication (e.g., improved food quality), the SC followed up to ask whether antibiotics would be effective for growth promotion. The sales agent’s initial and final recommendations were noted. 

In the disease treatment scenario, the SC explained that he or she has 15 chickens that appear to be ill (see Supplementary Materials Instrument S2), and that a neighbor recently used colistin for his chickens, which was very effective. The SC then asked to purchase colistin. The sales agent’s response constituted the initial recommendation. The SC then provided standardized additional information if requested. If the sales agent initially recommended a treatment other than medication for the chickens, the SC asked whether colistin would be an appropriate treatment. As in the first scenario, the sales agent’s recommendation was noted for the first inquiry and follow-up question.

The SCs concluded each interaction in both scenarios by thanking the sales agent and explaining that they could not make a purchase at that time. Immediately following the interaction, the SC walked out of view of the storefront to a private location and immediately completed a standardized survey questionnaire that described the sales agents’ recommendations, statements, and behaviors of interest. At the end of each day, completed questionnaires were given to the field coordinator, who subsequently coded the data and entered them into a spreadsheet for analysis.

### 4.2. Survey Procedures

Households were enrolled in this study if they met the following inclusion criteria: (1) there was a primary childcare provider present over the age of 18 years or legally emancipated; (2) there was a child present in the household between age 6 months to 5 years; (3) the household owned food animals; (4) consent was provided by a primary childcare provider to participate in the study. One hundred and seventeen households with small-scale food animals were recruited and enrolled into the study. 

The 2019 survey addressed a variety of topics related to food animal production and child health (note: child health outcomes were not included in this analysis). While small-scale production lacks a formal cutoff point regarding the number of animals produced, this study considers small scale animal producers as individuals or households who own fewer than 500 food animals. Producers of any type of food animal were included in this study. In addition to demographic information, survey questions measured animal ownership and the use of antibiotics in food animal production.

### 4.3. Data Analysis

Survey responses and records of simulated client interactions were converted to comma separated values (.csv) files and analyzed using R version 3.6.2 and R Studio version 1.1.383 (R Core Team, Austria). The average initial and final antibiotic recommendations were calculated and chi-square tests for independence were conducted to determine potential bivariate relationships between store characteristics and antibiotic recommendation practices. A chi-square test for independence tests whether two categorical variables are associated with one another, where a chi-square test statistic corresponding to a *p*-value less than 0.05 indicates that the two variables are associated with one another. The class of each antibiotic recommendation was analyzed to assess the proportion of antibiotic recommendations that belonged to each of the European Medicine Agency’s antibiotic categories for stewardship [27]. These categories include category A: “avoid” (antibiotics that are not authorized in veterinary medicine in the European Union; referred to as “highest caution”), category B: “restrict” (antibiotics that are critically important for human health and should be restricted for use in animals; referred to as “high caution”), category C: “caution” (antibiotics with few alternatives that should only be used when category D antibiotics would be ineffective; referred to as “medium caution”), and category D: “prudence” (antibiotics that should be used as first-line treatments; referred to as “low caution”) [27]. Investigators excluded instances where the sales agent recommended an antibiotic but did not recommend a specific antibiotic by name from the analysis of antibiotics by stewardship category.

## 5. Conclusions

The findings presented here suggest that households practicing small-scale food animal production are often encouraged to use antibiotics unnecessarily. Results from the novel simulated client method indicate that veterinary sales agents frequently recommend antibiotics for growth promotion and commonly recommend inappropriate classes of antibiotics for disease treatment. There was some evidence that antibiotic recommendation practices may be related to sales agent training. Few sales agents were willing to sell colistin, indicating the effectiveness of Ecuador’s recent ban despite its lack of enforcement. Given international efforts to promote small-scale food animal production in LMICs as a method of economic empowerment, proponents must consider traditional practices as well as new approaches to increase the prudent use of antibiotics and mitigate the public health impact of antibiotic use in small-scale food animal production systems. Finally, it is critical that supporters of small-scale production incorporate ABR considerations into their programs and policies in order to create alternatives to antibiotic use for small-scale food animal producers.

## Figures and Tables

**Table 1:** Survey results among study households that owned small-scale food animals.

Characteristics of Small-Scale Food Animal Producers	*n* (%)
*Number of food animals per household* (*n* = 117)	
1–10 animals	63 (54%)
11–20 animals	24 (20%)
21–30 animals	9 (8%)
31–40 animals	8 (7%)
41–50 animals	4 (3%)
>50 animals	9 (8%)
*Household owns chickens* (*n* = 117)	
Yes	94 (80%)
No	23 (20%)
*Number of chickens owned* (*n* = 94)	
1–10	73 (78%)
11–20	13 (14%)
>20	8 (8%)
*Household gave antibiotics to food animals in last 6 months* (*n* = 117)	
Yes	24 (21%)
No	93 (79%)
*Food animal species reported to be treated with antibiotics* (*n =* 24, *not mutually exclusive*)	
Poultry (chickens, ducks, and quail)	21 (88%)
Pigs	5 (21%)
Cows	4 (17%)
Other animals	9 (37%)
*Purchasing location for antibiotics* (*n* = 24)	
Veterinarian	18 (75%)
Animal products store	6 (25%)
*Instructions received for use of antibiotics* (*n* = 24)	
Written information	9 (38%)
Oral	10 (42%)
No information	2 (8%)
Other	3 (12%)

**Table 2:** Simulated client method participants in the growth promotion scenario and disease treatment scenario.

Characteristic	Growth Promotion Scenario (*n* = 38)		Disease Treatment Scenario (*n* = 40)	
	*n*	Proportion	*n*	Proportion
*Neighborhood*				
Checa	1	3%	1	3%
Pifo	10	26%	10	25%
Puembo	2	5%	4	10%
Quinche	8	21%	8	20%
Tumbaco	9	24%	9	22%
Yaruqui	8	21%	8	20%
*Store carried antibiotics*				
Yes	31	82%	33	83%
No	7	18%	7	17%
*Sales agent role*				
Veterinarian	3	8%	3	8%
Store Owner	14	37%	4	10%
Employee	21	55%	33	82%

**Table 3:** Proportion of sales agents’ initial and final recommendations by the two simulated client method scenarios.

Scenario	Recommendation	Initial Recommendation ^1^	Final Recommendation ^1^
*Growth Promotion Scenario* (*n* = 38, *not mutually exclusive*)			
	Antibiotics	37%	61%
	Medication (non-antibiotic)	76%	74%
	Veterinary consultation	11%	8%
	Improved food quality	42%	39%
*Disease Treatment Scenario* (*n* = 40, *not mutually exclusive*)			
	Antibiotics	75%	62%
	Medication (non-antibiotic)	40%	35%
	Veterinary consultation	22%	18%
	Improved food quality	35%	25%

^1^ The initial and final recommendations refer to the sales agents’ immediate recommendations to the simulated client and the recommendation following simulated client inquiry regarding the effectiveness of antibiotics, respectively. These values reflect the total proportion of sales agents that recommended each treatment in each scenario.

**Table 4:** Antibiotic recommendations by antibiotic stewardship category ^1^.

		Growth Promotion Scenario ^2^		Disease Treatment Scenario	
		*n*	Proportion	*n*	Proportion
*Encouraged antibiotic use as initial or final recommendation*	Yes	25	66%	33	83%
	No	13	34%	7	17%
*Highest stewardship category antibiotic recommended*	Highest caution	0	0	1	3%
	High caution	2	9%	7	21%
	Medium caution	4	18%	8	24%
	Low caution	16	73%	17	52%

^1^ These categories are based on the European Medicines Agency’s (EMA) categorization of antibiotic stewardship by antibiotic class. These categories include highest caution (EMA category A: avoid) antibiotics that are not authorized in veterinary medicine in the European Union, high caution (EMA category B: restrict) antibiotics that are critically important for human health and should be restricted for use in animals, medium caution (EMA category C: caution) antibiotics with few alternatives that should only be used when low caution antibiotics would be ineffective, and low caution (category D: prudence) antibiotics that should be used as first-line treatments [27]. ^2^ In the growth promotion scenario, three sales agents recommended antibiotics for disease treatment but did not recommend a specific antibiotic by name, so these three recommendations have been excluded from the subsequent analysis by antibiotic stewardship category.

**Table 5:** Bivariate relationships ^4^ between sales agents’ characteristics and antibiotic recommendation practices.

Sales Agent Characteristic	Recommendation Number	Growth Promotion Scenario		Disease Treatment Scenario	
		χ^2^ Test Statistic	*p*-Value	χ^2^ Test Statistic	*p*-Value
*Veterinarian present*	Initial	1.138	0.286	2.413	0.299
	Final	4.316	0.038 *	4.004	0.135
*Sales agent asked for more information*	Initial	0.17	0.68	0.139	0.709
	Final	0	1	1	0.317
*Sales agent appeared invested in the animals’ health*	Initial	0.005	0.945	0	1
	Final	0.398	0.528	0	1

* *p* < 0.05. ^4^ The given bivariate statistics describe the relationships between sales agents’ characteristics (yes vs. no) and antibiotic recommendation practices (recommended vs. did not recommend). A significant *p*-value (*p* < 0.05) indicates a relationship between the given sales agent characteristic and antibiotic recommendation practices.

## Supplementary Materials

The following are available online at https://www.mdpi.com/2079-6382/10/1/2/s1, Instrument S1: Simulated client guide for growth promotion scenario and Instrument S2: Simulated client guide for disease treatment scenario.
