# Supplementary material for: Assessing Upstream Determinants of Antibiotic Use in Small-Scale Food Animal Production through a Simulated Client Method
Source: Antibiotics (Basel). 2020 Dec 23;10(1):2. doi: 10.3390/antibiotics10010002 (PMC7822171; doi:10.3390/antibiotics10010002)
Supplement: Supplementary file 1 [file antibiotics-10-00002-s001.pdf]

Simulated Client Instruments

Instrument 1. SC Guide for Growth Promotion Scenario

"Client" walks into a pre-identified animal feed/veterinary supply store. Client approaches counter and explains that s/he has 25 chickens, and they are growing slowly. The client states that his/her neighbor's chickens seem to be growing much more quickly. The client wants advice.

If probed, client reports more detail on the situation. The chickens do not appear sick, but the client hopes to promote growth. 20 of these chickens are 1-2 years old, while 5 of these chickens are only 10 days old. The client presents the living conditions of the chickens, and will be able to provide additional information such as the chickens' size and normal diet. They should also be familiar with other animals that are being raised, as well as how long the chickens have been growing slowly.

- *If* ***first*** *offered non-medication advice (e.g., ensure chickens are hydrated, other non-medication related things), client then asks about antibiotics.*

  [CLIENT TAKES NOTES OF RECOMMENDATIONS BEFORE ANTIBIOTICS AND THEN PROBES FOR ANTIBIOTICS]

  - *If client asks about antibiotic and store does not carry antibiotic or vendor does not want to sell [NOTE REASON], client thanks vendor and leaves.*

- *If vendor offers non-antibiotic medication, client asks whether antibiotic would be helpful*

- *If offered antibiotic(s), client asks what it will do, what is in it, and how to use it*

  [CLIENT WILL NOTE IF S/HE HAS TO PROBE FOR THIS INFORMATION OR IF IT IS OFFERED UP-FRONT]

  - *Client asks about the cost of medication*
  - *Client asks about any risks of medication*
  - *Client explains that s/he does not want to buy anything at that time and leaves*

- *If vendor only offers antibiotics for the chickens that are 1-2 years old, client asks why vendor does not recommend antibiotic for 10-day old chickens.*

2. Simulated Client Guide for Disease Treatment Scenario

"Client" walks into a pre-identified animal feed/veterinary supply store. Client approaches counter and explains that s/he has 15 chickens, some of them appear to be sick. For the past week about 5 chickens have had diarrhea and their wings are drooping. They have not been eating. The client reports that his/her neighbor used colistin when his chickens were sick and they recovered quickly. The client asks if they can purchase colistin.

If probed, client reports more detail on the situation such as the age, size, normal diet, and living conditions of the chickens. They should also be familiar with other animals that are being raised, as well as if this was first noticed in one chicken and then spread, or all chickens appeared to get sick around the same time.

- *If **first** offered non-medication advice (e.g., ensure chickens are hydrated, other non-medication related things), client then asks about medication.*
  [CLIENT TAKES NOTES OF RECOMMENDATIONS BEFORE MEDICATION AND THEN PROBES FOR MEDICATION]
  - *If client asks about antibiotic and store does not carry antibiotic or vendor does not want to sell [NOTE REASON], client thanks vendor and leaves.*
- *If vendor offers other medication or antibiotic, client asks why recommended medication would be better than colistin.*
- *If offered colistin, client asks what it will do, what is in it, and how to use it*
  [CLIENT WILL NOTE IF S/HE HAS TO PROBE FOR THIS INFORMATION OR IF IT IS OFFERED UP-FRONT]
  - *Client asks about the cost of medication*
  - *Client asks about any risks of medication*
  - *Client explains that s/he does not want to buy anything at that time and leaves*
- *If vendor states that they do not carry colistin, client requests another last-line antibiotic (a third generation cephalosporin, a carbapenem, etc) [NOTE NAME OF DRUG REQUESTED]*
  - *If offered this drug:*
    - *Client asks about the cost of medication*
    - *Client asks about any risks of medication*
    - *Client explains that s/he does not want to buy anything at that time and leaves*
